# Downregulation of miR-199a-5p promotes prostate adeno-carcinoma progression through loss of its inhibition of HIF-1α

**DOI:** 10.18632/oncotarget.18315

**Published:** 2017-05-31

**Authors:** Jinjing Zhong, Rui Huang, Zhengzheng Su, Mengni Zhang, Miao Xu, Jing Gong, Ni Chen, Hao Zeng, Xueqin Chen, Qiao Zhou

**Affiliations:** ^1^ Department of Pathology, West China Hospital and National Key Laboratory of Biotherapy, Sichuan University, Chengdu 610041, China; ^2^ Department of Nuclear Medicine, West China Hospital, Sichuan University, Chengdu 610041, China; ^3^ Department of Urology, Institute of Urology, West China Hospital, Sichuan University, Chengdu 610041, Sichuan, China

**Keywords:** prostate cancer, microRNA, miR-199a-5p, HIF-1α

## Abstract

Hypoxia-inducible factor-1 alpha (HIF-1α) plays key roles in cell survival under both hypoxia and normoxia conditions. Regulation of HIF-1α is complex and involves numerous molecules and pathways, including post-transcriptional regulation by microRNAs (miRNAs). Although upregulation of HIF-1α has been shown to promote prostate adenocarcinoma (PCa) progression, the mechanism by which miRNAs modulate HIF-1α in prostate cancer has not been clarified. Here, we show that miR-199a-5p is underexpressed in prostate adenocarcinoma. Artificial overexpression of miR-199a-5p decreased cell proliferation, motility, and tumor angiogenesis and increased apoptosis in PCa cell liness PC-3 and DU145 by directly targeting the 3’-untranslated region (UTR) of HIF-1α mRNA, which reduced HIF-1α levels as well as downstream genes transactivated by HIF-1α (such as VEGF, CXCR4, BNIP3 and BCL-xL). Abnormalities of miR-199a-HIF regulation may contribute significantly to PCa pathogenesis and progression.

## INTRODUCTION

Prostate adenocarcinoma (PCa) is the most common non-skin cancer and a leading cause of cancer-related death in the United States and Europe [1]. PCa morbidity and mortality are also increasing dramatically in Asia, including China [2, 3]. In the past few decades, improvements in screening, diagnostics and treatment have led to a consistent decrease in PCa mortality and an increase in overall survival rates [4]. Nonetheless, hormone-refractory PCa (HRPCa), which is resistant to therapeutic modalities after an initial response to androgen deprivation, remains a significant challenge in prostate cancer treatment [5]. Although numerous genes are implicated in PCa, the molecular mechanisms underlying its progression remain poorly understood.

Hypoxia-inducible factor-1α (HIF-1α) is a trans-cription factor normally regulated by oxygen concentration but is often overexpressed in solid tumors such as cancers of the colon, breast, pancreas, kidney, prostate and bladder [6, 7]. Genes transactivated by HIF-1α include VEGF and erythropoietin (involved in angiogenesis and erythropoiesis), cyclin and FGF (which promote cell proliferation), GLUT-1 (participating in glucose metabolism), MDR1 (important for drug resistance), BCL2-xL (which suppresses apoptosis), and CXCR4 (inducing cell migration and invasion) [8–12].

Dysregulation of miRNA expression and function has been shown to be important for tumorigenesis and progression [13]. For example, underexpression of let-7c, miR-100, miR-145 and miR-218 in PCa was found to be associated with metastasis, and overexpression of miR-182 and miR-96 indicates a higher risk of progression [14, 15]. In our previous reports, we demonstrated that downregulation of miR-145, miR-200c, and miR-199b is significantly associated with PCa progression; in contrast, we found hypoxia-inducible factor (HIF-1α), which may be suppressed by miR-199b, was overexpressed in PCa and inhibited apoptosis via BCL2-xL transactivation [11, 16–18]. Expression of miR-199a-5p has been reported to be downregulated in certain tumors, including malignant testicular tumors, colorectal cancer and hepatocellular carcinoma, but upregulated in others, such as melanoma, gastric cancer and pancreatic adenocarcinoma [19–24], indicating tumor-type-specific regulatory mechanisms.

Microarray screening has indicated that miR-199a-5p is downregulated in PCa, yet its role in PCa is still unknown [25]. Here, we demonstrate that miR-199a-5p suppresses HIF-1α via post-transcriptional regulation of its mRNA and that underexpression of miR-199a-5p in PCa contributes to high HIF-1α levels and PCa progression.

## RESULTS

### miR-199a-5p was significantly downregulated in prostate cancer

**Figure 1:**
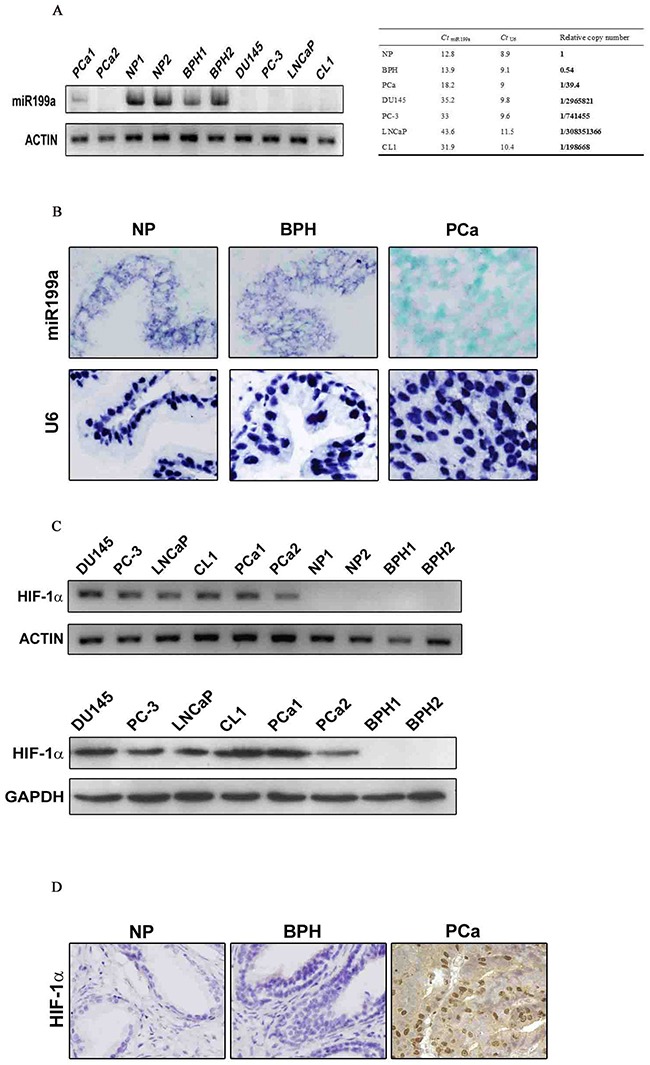
**(A)** Stem-loop RT-PCR (with actin as positive control) and Q-PCR (with U6 as internal control) analyses showing differential expression levels of mature miR-199a-5p in benign prostate tissue (NP and BPH) vs. prostate cancer (PCa) and prostate cancer cell lines DU145, PC-3, LNCaP and CL1. **(B)** Further validation of miR-199a-5p loss in prostate cancer by LNA-ISH (nuclear counterstaining with methyl green) with U6 as a positive control. miR-199a-5p and U6 signals are blue-purple (original magnification ×400). **(C)** In contrast to miR-199a-5p, HIF-1α mRNA (Figure 1C, upper panels, RT-PCR, the same actin control as in A) and protein (Figure 1C, lower panels, Western blot, GAPDH as positive control) levels were significantly higher in prostate cancer tissue and cells than in benign prostate tissue. **(D)** Immunohistochemical analysis of the HIF-1α protein in tissue sections. Nuclear and cytoplasmic positivity is in brown. Nuclear counterstaining with hematoxylin (original magnification ×400).

Expression of mature miR-199a-5p was investigated using stem loop RT-PCR. which showed high levels of miR-199a-5p expression were observed in benign prostate hyperplasia (BPH) and normal prostate (NP) tissue but very low levels in PCa tissue. miR-199a-5p was absent or barely detectable in DU145, PC3, LNCaP and CL1 cells (Figure 1A, left panel). Based on real-time Q-PCR, the level of miR-199a-5p in PCa tissue was only 1/39.4 (±0.000021) of that in benign prostate tissue, and it was undetectable in the four PCa cell lines (<1/10,000 of the level in benign prostate tissue; Figure 1A, right panel).

To further evaluate miR-199a-5p expression in tissue samples, the expression level of miR-199a-5p was evaluated by *in situ* hybridization (ISH) in 67 benign tissue samples and 51 PCa tissue samples (Figure 1B). The miR-199a-5p positivity rate in the benign prostate tissue samples (33/67, 49.3%) was significantly higher than that in the PCa tissue samples (15/51, 29.4%, *P*=0.038;).

### miR-199a-5p expression was inversely related to HIF-1α overexpression in prostate cancer

HIF-1α expression in fresh PCa tissue samples and cell lines was analyzed by RT-PCR and Western blotting, revealing high levels of both HIF-1α mRNA (Figure 1C) and protein (Figure 1D) in PCa tissue and cell lines, in contrast to their absence in NP or BPH samples.

Immunohistochemistry on tissue sections showed positive signals for HIF-1α in both the nucleus and cytoplasm, defined as HIF-N and HIF-C, respectively. The HIF-N (42/117, 35.9%) and HIF-C (21/117, 17.9%) positive rates were significantly higher than those in PCa in benign prostate tissue (0/67, 0%, both *P*=0.000; Table 2).

**Table 1:** Correlation between miR-199a-5p and HIF-1α expression and association of miR-199a-5p and HIF-1α expression levels with the clinicopathological characteristics of prostate adenocarcinoma

	miR199a-5p	HIF-C	HIF-N	AGE	Gleason	PSA
HIF-C	- 0.215					
HIF-N	- 0.393^**^	0.595^**^				
AGE	0.080	0.018	- 0.091			
Gleason	- 0.246	0.067	0.234^*^	-0.152		
PSA	- 0.006	0.208^*^	0.329^**^	0.048	0.089	
STAGE	- 0.259	0.117	0.370^**^	-0.134	0.310^**^	0.305^**^

HIF-C: Cytoplasmic immunostaining score of HIF-1; HIF-N: Nuclear immunostaining score of HIF-1; STAGE: TNM stage; PSA: Serum prostate-specific antigen; Gleason: Gleason score

*r*_s_: Spearman rank correlation coefficient; *P* values were determined by Fisher's exact test., ^**^, *P*<0.01, ^*^, *P*<0.05.

**Table 2:** Relationship of miR-199a-5p/HIF-1α expression levels with prostate cancer progression

	miR199a (+)	HIF-C (+) (%)	HIF-N (+) (%)
BPH	33/67 (49.3)^*^	0/67 (0.0)^*^	0/67 (0.0)^*^
PCa total	15/51 (29.4)	21/117 (17.9)	42/117 (35.9)
PCa with progression	5/21 (23.8)	8/58 (13.8)	27/58 (46.6)
PCa without progression	10/30 (33.3)	13/59 (22.0)	15/59 (25.4)
		*P* values	
BPH *vs.* PCa total	**0.038**	**0.000**	**0.000**
BPH *vs.* PCa with progression	**0.047**	**0.002**	**0.000**
BPH *vs.* PCa without progression	0.186	**0.000**	**0.000**
PCa with progression *vs.* without progression	0.543	0.336	**0.021**

Notes: miR-199a-5p(+) was defined as an ISH staining score of 4 or above. HIF-C(+) was defined as an IHC staining score of 2 or above. HIF-N(+) was defined as nuclear immunostaining score 3% or higher. *P* values were determined by Fisher's exact test.

Abbreviations: BPH, benign prostate hyperplasia; PCa, prostate cancer.

^*^ Number of miR-199a-5p(+) or HIF-1α(+) cases/total number of cases (positive rate %).

There was a significant inverse relationship between HIF-N and miR-199a-5p expression, as based on correlation analysis of the HIF-N protein level (determined by IHC) and miR-199a-5p level (determined by LNA-ISH) (*r*_s_=-0.393, *P*<0.01; Table 1). However, no significant correlation was found between HIF-C and miR-199a-5p (Table 1). The HIF-N protein level also correlated with the preoperative serum prostate-specific antigen (PSA) level, the Gleason score and the TNM stage. Conversely, miR-199a-5p expression alone was not significantly associated with PSA level, the Gleason score and the TNM stage (Table 1).

### Relationship of miR-199a-5p and HIF-N expression with tumor progression and PCa patient survival

In addition to the significantly different expression pattern of miR-199a-5p and HIF-1α in benign and cancerous prostate tissue samples (Tables 1 and 2; Figure 1), the positive rate of HIF-N in PCa with progression (27/58, 46.6%) was significantly higher than that in PCa without progression (15/59, 25.4%, *P*=0.021; Table 2).

**Figure 2:**
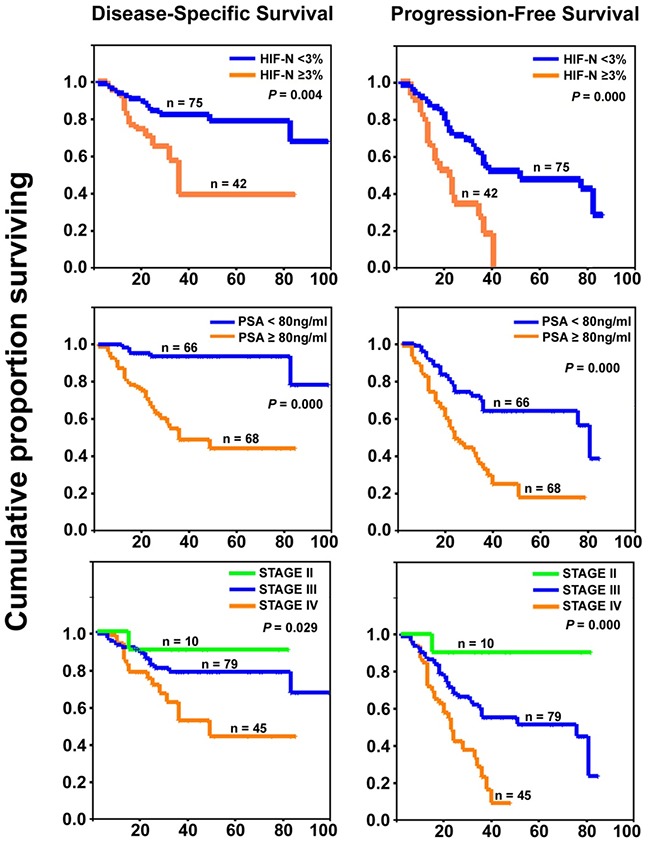
Prognostic significance of HIF-1α overexpression in prostate cancer Kaplan-Meier curves (with log-rank test *P* values) showing significantly poorer prognosis for patients with increased HIF-N, higher PSA level, and higher TNM stage. Abscissa: time in months.

Kaplan-Meier analysis showed an increased level of nuclear HIF-1α (HIF-N>3%) to be a significantly negative prognostic factor for both disease-specific survival (DSS) and progression-free survival (PFS) in PCa patients, as were the PSA level and TNM stage (*P*<0.05; Figure 2, Table 3). However, miR-199a-5p expression alone was not significantly associated with DSS or PFS (Table 3).

**Table 3:** Prognostic significance of miR-199a-5p/HIF-1α expression and clinicopathologic characteristics of PCa

	DSS		PFS	
	*n/N*	*P*	*n/N*	*P*
miR199a				
≥4	2/15	0.572	5/15	0.642
<4	8/36		16/36	
HIF-C				
≥2	5/21	0.920	8/21	0.701
<2	25/96		50/96	
HIF-N				
≥3	16/42	**0.004**	27/42	**0.000**
<3	14/75		31/75	
AGE				
≥70	11/48	0.395	26/48	0.734
<70	23/86		38/86	
Gleasonscore				
≥8	25/88	0.307	47/88	0.053
<8	9/46		17/46	
PSA				
≥80 ng/ml	29/68	**0.000**	42/68	**0.000**
<80 ng/ml	5/66		22/66	
STAGE				
II	1/10	**0.029**	1/10	**0.000**
III	16/79		33/79	
IV	17/45		30/45	

*P* value was determined by Kaplan-Meier survival analysis with the log-rank test.

DSS, Disease-specific survival; PFS, Progression-free survival. *n*: number of cases with events; *N*: total number of cases. miR199a: miR-199a-5p LNA-ISH score; HIF-C: cytoplasmic HIF1α immunostaining score; HIF-N: nuclear HIF1α immunostaining score.

### Identification of a potential HIF-1α 3’-UTR seed sequence

**Figure 3:**
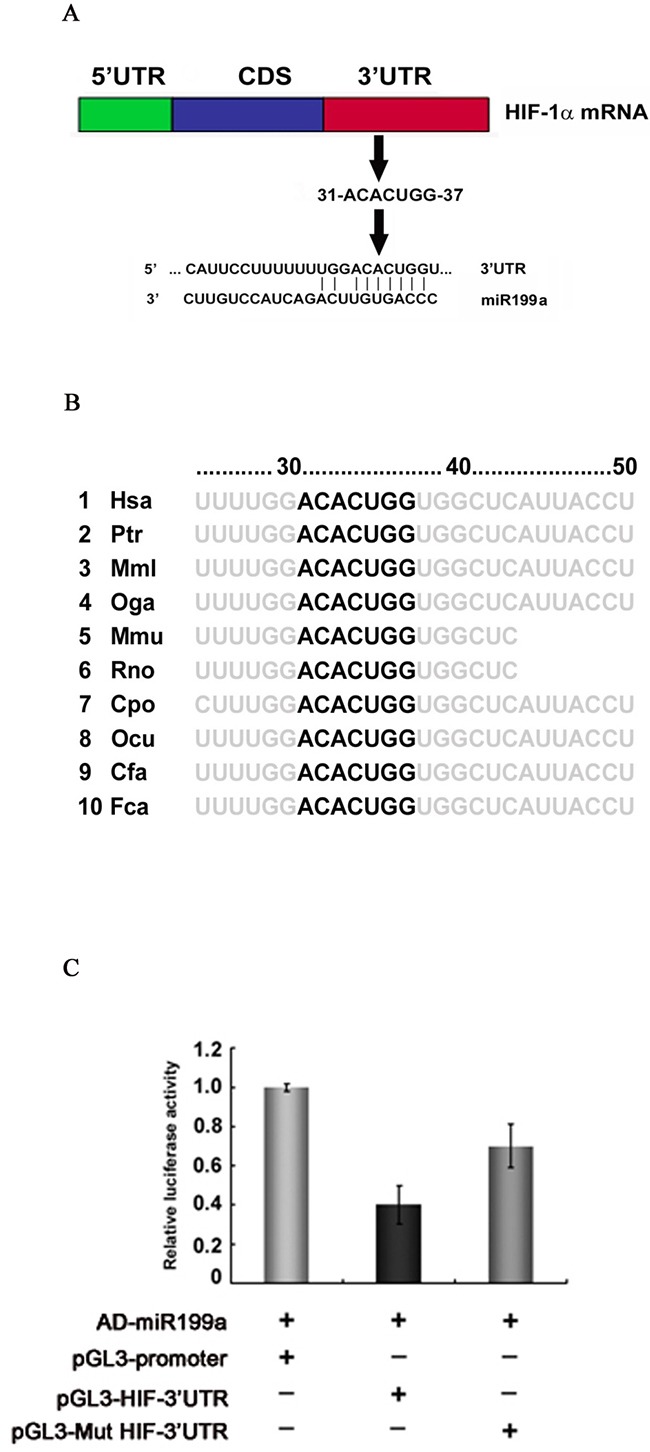
Identification of the miR-199a-5p seed sequence in the HIF-1α 3’-UTR and dual reporter gene assays for interaction between miR-199a and the HIF-1α 3’-UTR **(A)** The 31 to 37-nt sequence of HIF-1α was identified as a potential seed sequence for miR-199a-5p. **(B)** The seed sequence for miR-199a-5p in the HIF-1α 3’-UTR is conserved across species. **(C)** Dual reporter gene assays were performed using pGL3 expression constructs with HIF-1α 3’-UTR regions containing the seed sequence inserted downstream of the luciferase coding sequence; the activity of the basic pGL3 construct was used as the baseline (pGL3-Promoter). With artificial expression of miR-199a-5p (by coinfection with AD-miR-199a-5p), reporter gene activity, represented by relative luciferase activity (firefly/Renilla), was significantly decreased when the miR-199a seed sequence of the HIF-1α 3’-UTR was present (pGL3-HIF-3’UTR); mutation of the seed sequence (pGL3-Mut HIF-3’UTR) significantly restored reporter gene activity. Expression of miR-199a-5p alone had no effect on reporter gene activity when no seed sequence was present.

The 1174-nt 3’-UTR of the HIF-1α mRNA was analyzed using Target Scan 7.1 (http://www.targetscan.org), which identified miR-199a-5p as the major potential regulatory miRNA of HIF-1α. The 31 to 37-nt stretch of the HIF-1α 3’-UTR was the potential seed sequence (Figure 3A) highly conserved across species (Figure 3B). Sequence analysis showed no mutation or deletion of the 3’-UTR in PC-3, DU145, LNCaP or CL1 cells.

### miR-199a-5p targeted the 3’-UTR of HIF-1α

To demonstrate posttranscriptional regulation of HIF-1α mRNA by miR-199a-5p, luciferase reporter gene constructs were prepared. The potential seed sequence for miR-199a-5p and the flanking sequence of the HIF-1α 3’-UTR were cloned into luciferase reporter gene constructs. Constructs in which the seed sequences were mutated were also prepared.

With artificial overexpression of miR-199a-5p by infection with Ad-miR-199a-5p, dual reporter assays revealed significant downregulation of luciferase reporter gene activity by 62.1% (±0.012) for the pGL3-HIF-3’-UTR constructs. In contrast, luciferase reporter gene activity was largely restored when using the pGL3-Mut HIF-3’-UTR, in which the seed sequence of the HIF-1α 3’-UTR was mutated (Figure 3C).

### Artificial miR-199a-5p overexpression by adenoviral vectors led to downregulation of the HIF-1α protein and genes downstream of HIF-1α

**Figure 4:**
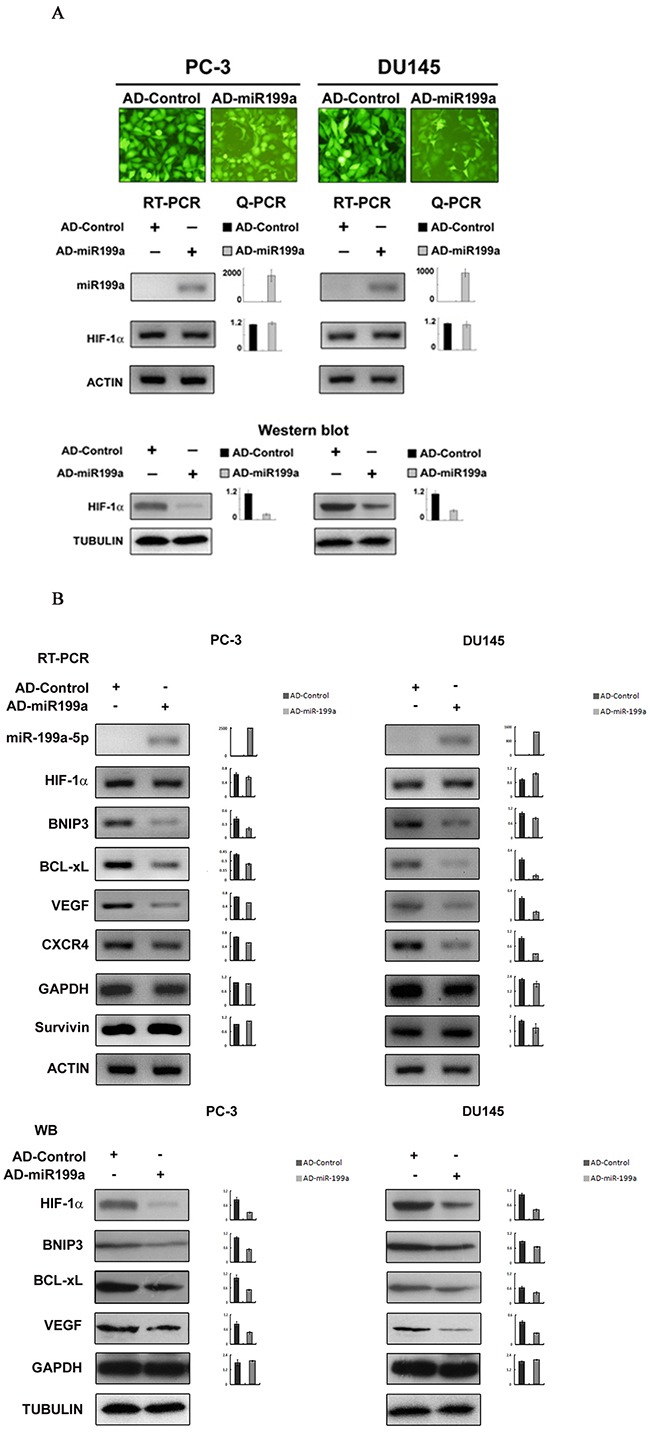
Effects on HIF-1α and HIF-1α downstream gene expression by artificial overexpression of miR-199a-5p in PC-3 and DU145 cells **(A)** The efficiency of AD-miR-199a and AD-Control infection is shown by homogeneous green fluorescence protein expression in infected cells (A, top panel, original magnification ×400). AD-miR-199a-5p-mediated miR-199a-5p overexpression led to no significant change in HIF-1α mRNA expression compared with AD-Control (A, middle panel, Q-PCR, mean±SD of three independent experiments, *P*>0.05), whereas significant overexpression of miR-199a-5p was observed (A, middle panel, Q-PCR, mean±SD of three independent experiments, *P*<0.05). AD-miR-199a-5p (but not AD-Control)-mediated miR-199a-5p overexpression led to downregualtion of the HIF-1α protein (A, bottom, Western blot with semiquantitative histograms, mean±SD of three independent experiments, *P*<0.05). **(B)** RT-PCR and Q-PCR shows that AD-miR-199a-5p (but not AD-Control)-mediated miR-199a-5p overexpression led to a decrease in expression of HIF-1α downstream genes (VEGF, CXCR4, BNIP-3 and BCL-xL) in PC-3 and DU145 cells. (top panel, histograms showing quantitative analysis of three independent experiments, *P*<0.05). Western blotting shows that the HIF-1α downstream genes BNIP3, BCL-xL, VEGF and CXCR4 were simultaneously downregulated upon artificial miR-199a-5p overexpression and consequent HIF-1α downregulation (bottom panel, histograms showing semiquantitative analysis of three independent experiments, *P*<0.05).

Concomitant with the artificial overexpression of mature miR-199a-5p by Ad-miR-199a infection, the HIF-1α protein level was significantly downregulated with miR-199a-5p though little change was observed for the level of HIF-1α mRNA (Figure 4A). Moreover, the HIF-1α downstream genes BNIP3, BCL-xL, VEGF and CXCR4 were also significantly downregulated artificial overexpression (Figure 4B).

### Biological effects of miR-199a-5p overexpression and HIF-1α downregulation

**Figure 5:**
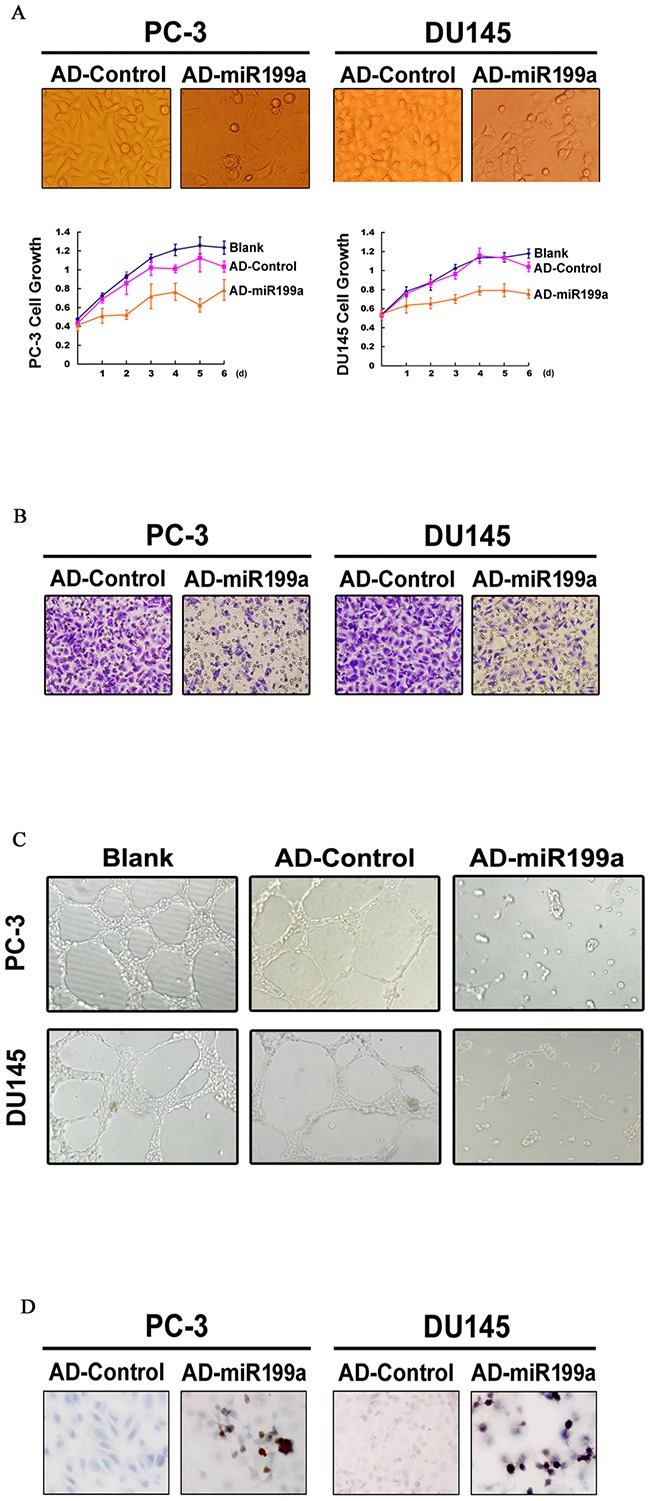
**(A)** Cell growth was significantly reduced upon artificial miR-199a-5p overexpression (original magnification ×400). **(B)** miR-199a-5p overexpression reduced cell invasiveness (original magnification ×400). **(C)** A tubule-formation assay showed reduced endothelial tubule formation when incubated with a tumor conditioned medium collected from PC-3/DU145 cells infected with AD-miR-199a (original magnification ×100). **(D)** Increased apoptosis upon miR-199a-5p overexpression, as shown by the TUNEL assay (original magnification ×400).

Synchronous with the changes in HIF/BNIP3/BCL-xL/VEGF/CXCR4 upon artificial miR-199a-5p overexpression, PC-3 and DU145 cells showed reduced growth, invasiveness and tumor angiogenic ability (Figure 5A, 5B and 5C) but increased apoptosis (as assessed by the TUNEL assay; Figure 5D).

## DISCUSSION

In this study, we show that HIF-1α mRNA is a functionally relevant target of miR-199a-5p, the downregulation of which contributes to the oncogenic high level of HIF-1α in Pca [11, 16–18], as well as tumor progression and unfavorable patient survival.

A large body of experimental evidence supports that hypoxia-mediated or -independent increases of HIF-1α plays critical roles in tumorigenesis and progression of many cancers via HIF-1α-dependent activation of genes that promote cancer cell survival or proliferation (such as BCL-xL), spread (e.g., CXCR4), and angiogenesis (e.g., VEGF) [26–29]. High levels of HIF-1α protein and mRNA have been observed in PCa tissues and cell lines [30–32], and our data demonstrate that HIF-N (nuclear HIF-1α expression, the functional state) correlates significantly with TNM stage, Gleason score, tumor progression and poor survival (both DSS and PFS).

HIF-1α, a transcription factor normally regulated by tissue oxygen concentrations [33], regulates numerous genes such as BNIP3, BCL-xL, VEGF, CXCR4, and Survivin [11, 34–37], which are involved in cell survival, proliferation, apoptosis and angiogenesis. Physiologically, HIF-1α mediates the cellular response to hypoxia. Under normoxic conditions, HIF-1α is hydroxylated at crucial prolyl residues, which is followed by binding to pVHL, ubiquitination and proteasome-mediated degradation. Conversely, hypoxia inhibits prolyl hydroxylation, thus preventing pVHL-mediated HIF-1α ubiquitination and degradation [38]. HIF-1α is regulated by major signaling pathways, including the extracellular signal-regulated kinase (ERK) and protein kinase B (AKT) pathways [39, 40]. More recent studies highlight the importance of epigenetic or post-transcriptional modulation of HIF-1α. For example, the histone deacetylase SIRT1 suppresses acetylation of H3K14 and inhibits HIF-1α at the transcriptional level [41]. The long non-coding RNA (lncRNA) CPS1-IT1 acts as a co-chaperone, alters Hsp90 and HIF-1α binding affinity and reduces HIF-1α activity in hepatocellular carcinoma [42]. In addition, LINK-A recruits BRK and LRRK2, which phosphorylate HIF-1α at Tyr565 and Ser797, respectively, preventing HIF-1α degradation [43].

In our previous work, we found reduced levels of miR-145, miR-200c and miR-199b, which target BNIP3, IRS1 and HIF-1α, respectively, in PCa [16–18]. A number of miRNAs that are differentially expressed in response to hypoxia (hypoxia-regulated miRNAs; HRMs) may stabilize/destabilize HIF-1α. Hypoxia-induced miR-210 and miR-424 may stabilize HIF-1α by repressing glycerol-3-phosphate dehydrogenase 1-like (GPDL1) and cullin2 (CUL2), respectively [44, 45]. Some HRMs such as miR-20b, miR-199 and miR-17-92 may destabilize HIF-1α [46–48]. We previously showed that miR-199b negatively regulated HIF-1α by targeting its 3’-UTR region and that downregulation of miR-199b in PCa induced cell growth and decreased cell death [17]. It has been reported that miR-199a-5p may also target HIF-1α to reduce proliferation and angiogenesis in melanoma, multiple myeloma and soft tissue sarcoma [49–51].

The miR-199a-5p expression status appears to vary in different tumors. For example, it has been reported to be upregulated in gastric cancer and pancreatic adenocarcinoma but downregulated in testicular cancer, ovarian cancer, multiple myeloma, colorectal cancer and hepatocellular carcinoma [19–24, 49, 52–54]. Different populations of cancer cells derived from epithelial ovarian cancers (EOC) may express different levels of miR-199a-5p [55, 56]. Therefore, miR-199a-5p expression appears to be defined by the specific tissue and environment.

Data for miR-199a-5p expression and functions in prostate cancer are very limited [25, 52, 57]. Two microarray screening assays indicate that miR-199a-5p is downregulated in PCa and that miR-199a-5p cooperates with miR-181a and miR-30d to reduce PCa drug resistance by targeting GRP78. Our data demonstrated that miR-199a-5p is downregulated in PCa, but, in contrast to HIF-1α nuclear expression level, it alone was not significantly associated with patient survival, indicating miR-199a-5p was among many factors influencing HIF-1α level, which was a major determinant of tumor progression and patient survival in PCa.

Several other genes have been shown to be targeted by miR-199a-5p [20–22, 52, 54, 58, 59]. Ets-1, V-ets erythroblastosis virus E26 oncogene homolog 1, is suppressed by miR-199a-5p, the loss of which may promote breast cancer cell invasion [59]. Inhibition of pro-cell proliferation gene CAC1 by miR-199a-5p reduces colorectal cancer cell growth and multidrug resistance [20]. DDR1, a collagen tyrosine kinase, is downregulated by miR-199a-5p, which decreases multiple myeloma cell invasiveness [49]. Nonetheless, it has been reported that miR-199a-5p induces melanoma metastasis and angiogenesis by inhibiting ApoE, an inhibitor of metastasis and angiogenesis, through interaction at distinct ApoE receptors [22]. These results indicate complex and tissue-specific regulatory differences of miR-199a-5p.

Little is known regarding the molecular mechanisms responsible for miR-199a-5p downregulation in PCa. It has been reported that the transcription factors TWIST1 and EGR1 as well as the methylation status of miR-199a promoters may be involved [60–62]. Activation of the AKT pathway, which is associated with PCa progression, can also downregulate miR-199a-5p [49, 63]. It is also intriguing that double-negative feedback loops exist between miR-199a-5p and its targets: miR-199a-5p targets and reduces expression of the Brm subunit of SWI/SNF, which negatively regulates Egr1 (a positive regulator of *miR-199a-2*, the precursor gene of miR-199a-5p) [61]. Clearly, further studies regarding the regulation of miR-199a-5p expression are needed.

In summary, we report that miR-199a-5p plays a tumor-suppressive role by directly targeting HIF-1α and thereby suppresses genes downstream of HIF-1α, such as VEGF, CXCR4, BNIP3 and BCL-xL. These data provide evidence for an additional dimension of the already rather complex regulation of HIF-1α in PCa, which may help in the development of miR-199a-5p-based strategies for treating this disease.

## MATERIALS AND METHODS

### Cell lines and general reagents

Human PCa cell lines PC-3, DU145, LNCaP and CL1 were obtained from American Type Culture Collection and maintained in RPMI 1640 medium (61870044; GIBCO, Rockville, MD) supplemented with 10% fetal calf serum (FCS, 1009-141; GIBCO, Rockville, MD). The adenovirus-immortalized human embryonic kidney epithelial cell line HEK-293 was maintained in Dulbecco's Modified Eagle Medium (DMEM, 10566016; GIBCO, Rockville, MD) with 10% FCS. Tris base, Tween 20, dithiothreitol (DTT), and ethylenediaminetetraacetic acid (EDTA) were purchased from Amresco (Solon, OH). Phenylmethylsulfonyl fluoride, leupepstatin, and aprotinin were purchased from Roche Diagnostics (Mannheim, Germany).

### Tissue samples and clinical data

203 archived, formalin-fixed, paraffin- embedded samples, including 134 prostate adeno-carcinomas (121 needle biopsies, 13 transurethral resections of prostate samples) and 69 benign prostate tissues (all from needle biopsies), were used. In addition, 6 snap-frozen fresh tissue samples (2 cancerous, 2 benign prostate hyperplasia and 2 normal) obtained from prostatectomy specimens were included. All tissue samples were obtained from West China Hospital and were collected and used according to the ethical guidelines and procedures approved by the institutional supervisory committee. Exclusion criteria for the prostate adenocarcinoma group were the presence of another cancer or previous treatment (surgery, endocrine therapy or chemo-radiotherapy). The Gleason scores (GS) of the prostate adenocarcinomas were as follows: GS6 (8 cases, 6%); GS7 (38 cases, 28%); and GS8-10 (88 cases, 66%). The tumor-node-metastasis (TNM) stages were as follows: stage II, 10 cases (7%); stage III, 79 cases (59%); and stage IV, 45 cases (34%). This cohort of patients ranged in age from 53 to 86 years (mean, 69.2) and was treated by combined androgen blockade (surgical castration plus flutamide). Patients were followed by clinical and laboratory monitoring on a regular basis starting at definitive diagnosis. The disease-specific survival (DSS)time was defined as the time from definitive diagnosis to disease-specific death, and the progression-free survival (PFS) time was defined as the time from definitive diagnosis to any of the following events after initial treatment: prostate-specific antigen failure, local progression, metastasis, or disease-specific death.

### Stem-loop reverse transcription, conventional reverse transcription-PCR, and genomic DNA PCR

Total RNA was extracted using Trizol reagent (15596018; Invitrogen, Carlsbad, CA). The stem-loop reverse transcription-polymerase chain reaction (RT-PCR) technique was used to examine mature miR-199a-5p. The sequence of the stem-loop RT primer was 5’-GTCGTATCCAGTGCAGGGTCCGAGGTATTCGCACTGGATACG ACGAACACAGA-3’. RT-PCR was performed in a 20-μl volume containing 2.5 μg total RNA, 1.6 mmol/l miR-199a stem-loop primer, 2 μl 10 mmol/l deoxynucleotide triphosphates, 1 μl 0.1 mol/L DTT, and 1 μl M-MLV reverse transcriptase (2641Q; Takara, Dalian, China) at 16°C for 30 min, 42°C for 30 min, and 85°C for 5 min. The sequence of the PCR primers for mature miR-199a-5p were as follows: sense, 5’-GCATAGCCCGCCCAGTGTT-3’; antisense, 5’-GTGCAGGGTCCGAGGT-3’ (product length, 67 bp).

The random RT primer 5’-(dN)_9_-3’(D6045; Takara, Dalian, China) was used for other genes, the PCR primers of which were designed according to their respective cDNA sequences (GenBank), as follows: HIF-1α, 5’-CCTATGACCTGCTTGGTGCTG-3’ and 5’-CTGGCTCATATCCCATCAATTCG-3’, 157 bp; Actin, 5’-TGGAGAAATCTGGCACCAC-3’ and 5’-GAGGCGTACAGGGATAGCAC-3’, 190 bp; U6, 5’-TGGAACGATACAGAGAAGATTAGCA-3’ and 5’-AACGCTTCACGAATTTGCGT-3’, 66 bp; BNIP3, 5’-ACCAACAGGGCTTCTGAAAC-3’ and 5’-GAGGGTGGCCGTGCGC-3’, 159 bp; BCL-xL, 5’-CTGTGCGTGGAAAGCGTAG-3’ and 5’-CTCG GCTGCTGCATTGTTC-3’, 159 bp; Survivin, 5’-GCAG TTTGAAGAATTAACCCTTG-3’ and 5’-CACTTTCT CCGCAGTTTCCTC-3’ 121 bp; VEGF, 5’-AGGAGGG CAGAATCATCACG-3’ and 5’-GCACACAGGATGGCT TGAAGA-3’, 140 bp; CXCR4, 5’-ACTACACCGAGGAA ATGGGCT-3’ and 5’-CCCACAATGCCAGTT AAGAAGA-3’, 133 bp; GAPDH, 5’-CCACCCATGGCAA ATTCCATGGCA-3’ and 5’-TCAAGACGGCAGG TCAGGTCCACC-3’, 597 bp. Standard PCR protocols were used. Products were resolved by 2% agarose gel electrophoresis or 10% polyacrylamide gel electrophoresis (PAGE) and visualized by staining with the fluorescent dye Goldview (HGV-II; SBS, Beijing, China). Images were captured using a Bio-Rad Gel Doc XR (Bio-Rad, Berkeley, CA). Semiquantitative analysis was performed using ImageQuant 5.2 software (Molecular Dynamics, WA).

The HIF-1α 3’-UTR was amplified and sequenced using the following primers: 5’-TCTAGACTCGAGTACAAGGCAGCAGAAAC-3’ and 5’-TCTAGAGTTTGTGCAGTATTGTAGCC-3’ (187 bp).

### Real-time quantitative PCR

Real-time quantitative PCR (Q-PCR) was used together with stem-loop RT to quantitate mature miR-199a-5p. Q-PCR was performed with Light Cycler software 4.05 (Roche Diagnostics, Mannheim, Germany), as described. The β-actin gene was used as a control. The copy number of target genes (relative to β-actin) was determined using the 2^−ΔΔCt^ method: ΔΔCt=ΔCt_exp_−ΔCt_con_=(Ct_exp-target_−Ct_exp-actin_)−(Ct_con-target_−Ct_con-actin_), where ‘exp’ represents the experimental group, ‘con’ the control group, and ‘target’ the gene of interest.

### Locked nucleic acid (LNA) in situ hybridization

A digoxigenin-labeled, LNA-modified miR-199a probe (20 nmol/L; 5’-CAACAGGTAGTCTGAACACTGG G-3’, Invitrogen) was incubated at 53°C for 18 h; an LNA probe for U6 (5’-CACGAATTTGCGTGTCATCCTT-3’) was used as a control (hybridization temperature at 50°C). The detailed procedure was described previously [16].

The locked nucleic acid *in situ* hybridization (LNA-ISH) signal intensity was recorded semiquantitatively, with 0 indicating no signal and 1, 2 and 3 indicating weak, moderate, and strong signals, respectively. The extent of LNA-ISH signal was defined as the percentage of cells showing signal and recorded as 0 (0%), 1 (1-30%), and 2 (>30%). An integrated score of LNA-ISH signal (obtained by the product of the intensity score and extent score) of 4 or more was designated as miR-199a-5p positive.

### Western blotting

Primary antibodies against the following were used: HIF-1α (mouse monoclonal, 1:1 500, MAB5382; Chemicon Inc, Billerica, MD); BNIP3 (mouse monoclonal, 1:3000, B7931; Sigma, St. Louis, MI); BCL-xL (rabbit polyclonal, SAB3500349; 1:1000, Sigma, St. Louis, MI); VEGF (rabbit polyclonal, 1:500, PB0084; Boster, Wuhan, China); GAPDH (mouse monoclonal, 1:10000, KC-5G4; KangCheng, Shanghai, China); and β-tubulin (mouse monoclonal, 1:1000, A06868; Boster, Wuhan, China). Horseradish peroxidase-labeled secondary antibodies were obtained from Zymed Laboratories Inc (San Francisco, CA). Western blot assays were performed as previously described [16].

### Immunohistochemistry

An anti-HIF-1α antibody used for immuno-histochemistry (mouse monoclonal, 1:200, MAB5382; Chemicon, Billerica, MD). Immunostaining was performed as previously described [11]. The percentage of cells with positive nuclear (HIF-N) or cytoplasmic (HIF-C) immunostaining was examined by light microscopy following a protocol we previously described [64]. The cut-off values for HIF-N and HIF-C were 3% and score 2, respectively, as based on preliminary assays.

### Recombinant adenoviral vectors for overexpression of miR-199a-5p

The pri-miR-199a (total sequence 422 bp) was amplified from HEK-293 cell genomic DNA with the indicated primers. The PCR product was cloned into pMD18-T (6011; TaKaRa, Dalian, China), verified by sequencing, and subcloned into the shuttle plasmid pAdTrack-CMV (designated pAdTrack-miR-199a). pAdTrack-miR-199a was linearized with PmeI and used to transform BJ5183-AD-1 cells harboring adenoviral pAdeasy-1 vectors (VXS0387; Stratagene, La Jolla, CA) for homologous recombination. Colonies were screened by plasmid miniprep and PacI restriction analysis to obtain clones with recombinant miR-199a (designated as AD-miR-199a). AD-miR-199a was amplified by repeated infection and verified by PCR. The pAdTrack-CMV empty vector was used as a control (designated as AD-Control). Titers and multiplicity of infection (MOI) values were determined according to the manufacturer's protocols.

### Cell viability assay

Cells were collected and stained with trypan blue (200 mg/ml, T6146; Sigma, St. Louis, MO). The number of viable cells was determined by microscopic examination.

### MTT [3-(4,5-dimethylthiazol-2-yl)-2,5-diphenyl-tetrazolium bromide] assay

PC-3 and DU145 cells were incubated overnight in a 96-well plate (1×10^4^ cells/ml) and then treated with AD-miR-199a. Cells incubated with AD-Control served as the control group. Cell viability was evaluated at 24 h, 48 h, and 72 h of incubation using the MTT (298-93-1; Amresco, Solon, OH) assay. The MTT assays were performed using a standard protocol, and the optical density was measured at 570 nm using a spectrophotometer. The background wavelength of MTT is 630 nm.

### Terminal deoxynucleotidyltransferase-mediated biotinylated dUTP nick-end labeling

Terminal deoxynucleotidyltransferase-mediated dUTP nick-end labeling (TUNEL) was performed using an *in situ* cell death detection kit (11 684 817 910; Roche Diagnostic, Mannheim, Germany), as previously described [11].

### Transwell invasion assay

AD-miR-199a- or AD-Control-infected PC-3 and DU145 cells (1.0×10^5^ cells) were seeded in Matrigel-coated chambers with 8-μm pores (PIEP12R48; Millipore, Billerica, MA). Cells were suspended in serum-free medium and allowed to migrate toward a complete medium supplemented with 10% fetal bovine serum for 36 h. Non-invading cells were physically removed by scraping, and invading cells were stained with 0.1% crystal violet solution and counted under a light microscope.

### Luciferase reporter constructs and site-directed mutagenesis

The seed sequence of the HIF-1α 3’-UTR (31-37 nt) and flanking sequences were amplified from PC-3 cell genomic DNA. UTR-wt (-65 bp to +113 bp, with +1 being the first base after the stop codon) was prepared with the primers HIF-1α-XbaI-FP (5’-TCTAGACTCGAGTACAAGGCAGCAGAAAC-3’) and HIF-1α-XbaI-RP (5’-TCTAGAGTTTGTGCAGTATTGTAGCC-3’). The PCR products were cloned into pMD18-T and subcloned into pGL3-Promoter (E1761; Promega, Madison, WI); the product was designated pGL3-HIF-3’UTR. Overlapping PCR was used for site-directed mutagenesis of the seed sequence (from ACACTGG to CAGATCT, designated pGL3-mut HIF). The PCR primers were HIF-UTR-MUT-FP (5’-GGCAGATCTTGGCTCACTACC-3’) and HIF-UTR-MUT-RP (5’-CAAGATCTGCCAAAAAAAGGAATG-3’).

### Dual reporter gene assay

PC-3 cells were cultured in 24-well plates and transfected with 0.4 μg of the reporter constructs using Lipofectamine 2000 (11668019; Invitrogen, Carlsbad, CA). The pRL-CMV plasmid (E2261; Promega, Madison, WI) containing the *Renilla* luciferase gene (0.02 μg) was cotransfected as an internal control. Cells were infected with AD-miR-199a or AD-Control (MOI 100) at 4 h after transfection and collected 24 h later. Firefly and *Renilla* luciferase activities were assessed using a Luminometer TD-20/20 (Turner Design, Sunnyvale, CA).

### Tubule-formation assay to measure *in vitro* angiogenesis of vascular endothelial cells

Culture plates (48-well) were coated with 100 μl of Matrigel (356234; BD, San Jose, CA) per well and incubated at 37°C for 1 h or until the gel solidified. A total of 5×10^4^ human umbilical vein endothelial cells (HUVECs) were then seeded in each coated well and incubated with a tumor conditioned medium. The tumor conditioned medium was collected from cultures of PC-3 or DU145 cells that had been infected with AD-miR-199a or AD-Control for 48 h. After incubation at 37°C for 2, 4, 6, 8, 12, or 24 h, the HUVEC cells were examined for capillary-like network formation and imaged under a light microscope for each time point. Images were captured from 5 different fields in each well. Tubule formation was quantified by measuring the total tubule length and total number of branch points in triplicate wells using Image Pro Plus 6.0 (Media Cybernetics, Rochville, MD) software according to a published protocol [65].

### Statistical analyses

The SPSS 10 program was used for general statistical and survival analyses. Fisher's exact test was used for between-group comparison, and Spearman rank order correlation was used for correlation analysis. The Kaplan-Meier method with the log-rank test was used for univariate survival analysis.

## Abbreviations

BCL2-xL: BCL2 Like 1
BNIP3: BCL2/Adenovirus E1B 19kD Interacting Protein 3
BPH: benign prostate hyperplasia
CXCR4: C-X-C chemokine receptor type 4
HIF-1α: hypoxia-inducible factor-1α
miRNA: microRNA
PCa: prostate adenocarcinoma
VEGF: vascular endothelial growth factor
